# Fast prototyping of memristors for ReRAMs and neuromorphic computing

**DOI:** 10.1039/d5nr02690c

**Published:** 2025-12-10

**Authors:** Gianluca Marraccini, Sebastiano Strangio, Elisabetta Dimaggio, Riccardo Sargeni, Francesco Pieri, Yigit Sozen, Andres Castellanos-Gomez, Gianluca Fiori

**Affiliations:** a Dipartimento di Ingegneria dell'Informazione, Università di Pisa, via G.Caruso 16 Pisa Italy gianluca.marraccini@ing.unipi.it; b Department of Electrical, Computer and Biomedical Engineering, Università di Pavia Pavia Italy; c Quantavis s.r.l., Largo Padre Renzo Spadoni Pisa Italy; d 2D Foundry research group, Instituto de Ciencia de Materiales de Madrid (ICMM-CSIC) Madrid E-28049 Spain

## Abstract

The growing demand for energy-efficient computing in artificial intelligence requires novel memory technologies capable of storing and processing information. Memristors stand out in thanks to their ability to store information, mimic synaptic behavior and support in-memory computing architectures while requiring minimal active areas and energy consumptions. Here is presented a scalable and cost-effective approach to fabricate Ag/MoS_2_/Au memristors as resistive switching memory devices by combining roll-to-roll mechanical exfoliation of two-dimensional materials with inkjet printing. These devices exhibit reliable non-volatile switching behavior attributed to the formation and dissolution of metallic conductive filaments within the MoS_2_ layer, with high resistance ratios and robust retention times. A fully-connected neural networks is simulated using quantized weights mapped onto a virtual memristor crossbar array demonstrating that classification tasks can be performed with high accuracy even with limited bit-width precision, highlighting the potential of these devices for energy-efficient, high-throughput AI hardware.

## Abstract 2

This work presents a scalable method to create Ag/MoS_2_/Au non-volatile memristors using roll-to-roll exfoliation and inkjet printing. Simulation shows that the weights of a Neural network can be stored in an array of the proposed devices.

## Graphical abstract

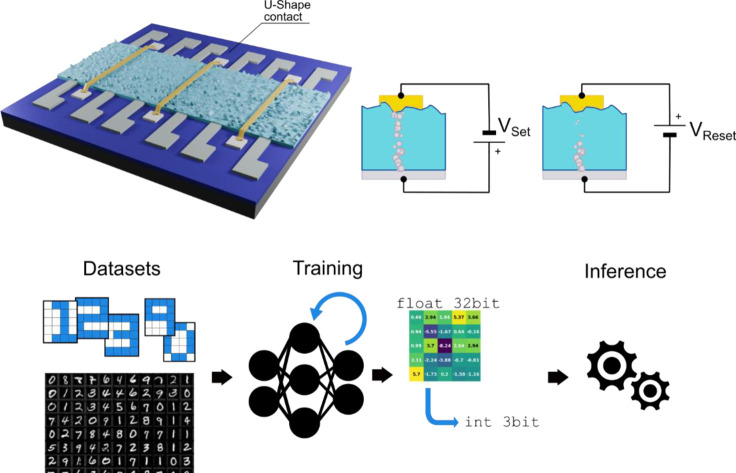

## 1 Introduction

Random Access Memories (ReRAMs) are a well-established class of non-volatile memory devices in which the basic memory cell consists of a resistive switching element capable of changing its conductance in response to an external electrical stimulus. This effect is typically achieved through abrupt conductance changes triggered by voltage pulses applied across the device terminals. Since their initial study in the 1960s,^1^ memristive devices have gained significant attention due to their simple structure, their potential for high integration density, and their use in non-Von Neumann architectures.^2^ Over the years, several resistive switching mechanisms have been discovered and investigated.^3–6^ Among these, phase-change memristors rely on a reversible transition between distinct structural phases, such as crystalline to amorphous lattice (as seen in doped GeSbTe).^7^ In these materials, thermally activated crystallization induces a lattice rearrangement from the amorphous phase to a crystalline phase, while a return to the amorphous state is achieved by melting and abruptly cooling the material.

Another class are conductive filament (CF)–based memristors, which operate through the drift of metal ions under an applied electric field. This can cause either the formation or the rupture of conductive paths between the device terminals.^8–11^ which eventually leads to a low resistive state (LRS) or a high resistive state (HRS), respectively. The simplest structure for CF memristors is characterized by two metal electrodes separated by a switching layer (SL) material The two electrodes are usually made of metals (*e.g.*, copper or silver), noble metals (platinum or gold) or carbon-based materials, and they can either contribute to the switching phenomenon or simply act as current conductors. The SL is the material layer where the conductance switch takes place. The most common materials for the SL are oxides (*e.g.* TiO_*X*_, AlO_*X*_),^12–14^ perovskites or two-dimensional materials, such as Transition Metal Dichalcogenides (TMDs).^15–18^ When the SL is only a few atomic layers thick, Schottky emission and direct tunneling effects become the dominant transport mechanisms. In these cases, the HRS is primarily governed by thermionic current, while the LRS is characterized by tunneling current. This transition can be identified through the temperature-dependent current behavior, where the Schottky effect current equation 
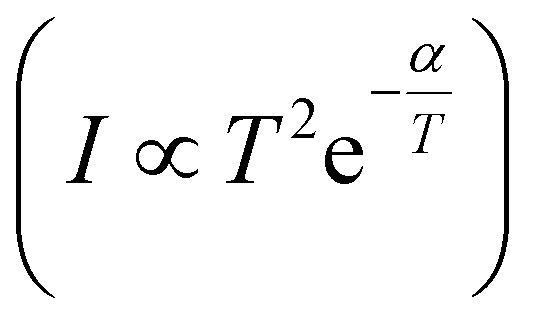
 is the best fitting curve of experimental data, whereas direct tunneling exhibits an inverse relationship with temperature.^19^ An interesting application of two-dimensional material-based memristors can be found in neuromorphic networks and architectures, where they show promising characteristics, such as gradual conductance changes, which mimic biological synapses and neural plasticity.^20,21^ For this purpose, both volatile^22^ and non-volatile^23–26^ memristors have been extensively employed. Volatile memristors are used for Spiking Neural Networks (SNNs),^27^ which encode information in pulse timing and rate. On the other hand, non-volatile memristors are suitable for multi-time programmable Read-Only Memory (ROM) applications, as they offer extended data retention, ranging from hours to years (demonstrated in simulations).^26^ An intriguing feature of the latter devices is their ability to support multiple stable resistance states,^28,29^ enabling multi-bit storage within a single cell or even analog data representation. This property has generated considerable interest in the research community, as it allows for the direct implementation of analog dot-product operations through memristor crossbar arrays.^30^ Such operations are fundamental for neural network processing and could significantly reduce the area and energy requirements associated with conventional digital multipliers.

In this work, a memristor with two stable resistance states was fabricated using a low-cost, high-throughput approach. The device consists of inkjet-printed silver and gold contacts on the top and bottom sides, respectively, of a semiconducting MoS_2_ layer, which is deposited *via* a roll-to-roll technique.^31^ Electrical characterization confirmed that the device can be electrically switched between high- and low-resistance states. It is supposed that when a positive voltage is applied to the silver contact, silver ions migrate towards the gold electrode, leading to the formation of a conductive filament within the exfoliated MoS_2_, following the mechanism demonstrated by the work of Yang *et al.*^8,32^ Conversely, applying a reverse voltage is presumed to dissolve the filament, restoring the high-resistance state. Electrical measurements were performed to derive the current–voltage characteristic of the devices and extract the key parameters and their statistical distribution.

These parameters were then used to simulate a neural network using a large-scale memristor-based crossbar array. In the simulation, a deep neural network was trained, and its parameters (*i.e.*, weights and biases) were quantized from floating-point to signed integer, so that they could be implemented using our memory-cell devices. This quantization reduced the bit-width required for storage and the corresponding processing complexity, enabling efficient deployment in the simulated memristor array.

Simulation results showed that, for a simple dataset consisting of schematic representations of digits 0 to 9, an accuracy of 100% was achieved using 3-bit parameters in a fully connected neural network with three layers (*i.e.*, parameter values ranging from −4 to 3). When applied to the more complex Modified National Institute of Standards and Technology (MNIST) dataset, a 94% accuracy was obtained with 4-bit precision, requiring an architecture with four layers and a higher number of neurons.

## 2 Experimental

**Fig. 1:**
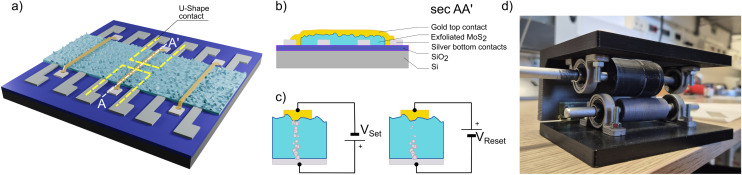
(a) Strip of exfoliated MoS_2_ sandwiched between a silver bottom and a gold top contact, making an isolated “crossbar array” of memristors. (b) Cross section of a device with Ag/MoS_2_/Au structure (c) working principle of a memristor. A positive bias voltage let a conductive filament grow and short the two contacts, an opposite voltage let the filament retract and a higher resistance state is reached. (d) Photo of the two rollers used to carry out the high throughput mechanical exfoliation.

The structure of the proposed vertical memristors is shown in Fig. 1a and b. It consists of a stacked configuration, where the bottom contacts (BC) is composed by a conductive line oriented perpendicular to an overlapped top contacts (TC) separated by a SL in between. The CF is expected to form within the SL under an electrical stimulus, as shown in Fig. 1c.

The SL in this work is obtained through the roll-to-roll process, first demonstrated by some of the authors of the present work^31^ and shown in Fig. 1d. In particular, a molybdenite crystal is first mechanically exfoliated onto a Nitto tape and, after the roll-to-roll process, a high density distribution of mechanically exfoliated MoS_2_ nanosheets on the tape is obtained. These nanosheets are then transferred onto an acceptor substrate by placing the tape in contact with the surface and heating it at 110 °C for 5 minutes. To enhance the transfer of MoS_2_ flakes from the tape to the substrate, it is essential that the target substrate is very clean. To ensure cleanliness, the SiO_2_ substrate undergoes a sequential cleaning process: first, a 5 minute sonication in Acetone (ACE) removes the major contaminants, followed by a rinse with Isopropilic Alcohol (IPA) to eliminate residues. Finally, the substrate is treated in a UV cleaner for 10 minutes before the MoS_2_ transfer step.

**Fig. 2:**
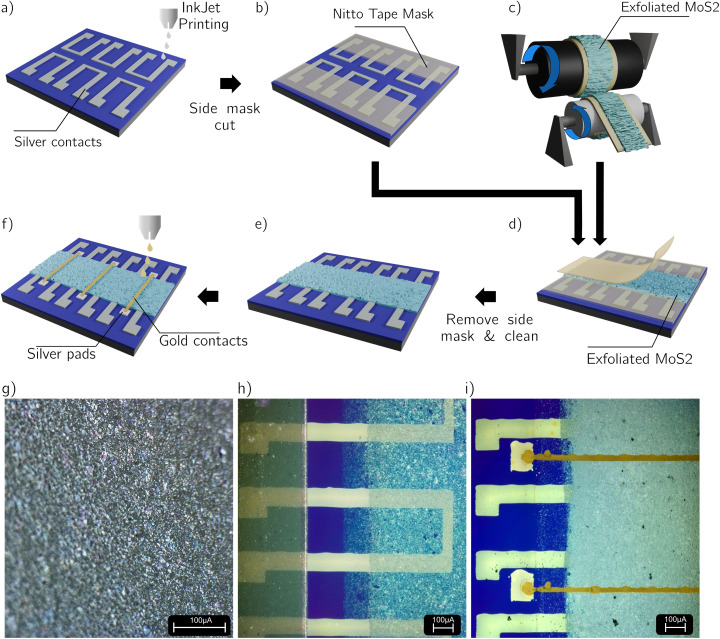
Main fabrication process steps of the Vertical memristors. (a) Silver BCs are inkjet printed with a silver nanoparticle ink. The area where the two contacts are ovrelapped is ∼60 μm × 60 μm. (b) The BC pads are covered by a hand-cut mask made of Nitto tape to avoid coverage by MoS_2_ flakes. (c) With the Scotch-tape exfoliation method, bulk flakes of MoS_2_ obtained by a molybdenite crystal are first positioned on the Nitto tape surrounding the two rollers. By spinning the two cylinders together the bulk flakes spread over the tape, and a uniformly MoS_2_ covered area is obtained. (d) A few strips are cut from this tape and transferred on the printed BC, following the thermal release transfer described in the text. This step is repeated until the BC is fully covered. (e) The pad mask is removed and the batch is cleaned in acetone (heated at 40 °C); acetone residues are then removed with a quick bath in IPA the batch is dried on a hot plate at 110 °C. (f) Gold TCs, with additional lateral silver pads for external electrical contact, are finally printed. (g) Optical photo of the exfoliated MoS_2_ on Nitto tape after the step in panel c. (h) Optical photo of the silver BC covered with the Nitto Mask and the first transfer of the semiconductor. (i) Optical photo of the completed devices, with the gold TC crossing the vertical part of the BC.

When the substrate is clean, silver BCs are Inkjet printed in a crossbar array. In order to keep each BC pad separated from the MoS_2_ area, they are printed with a “U-shape” design. The BCs horizontal lines measure 2 mm × 100 μm, while the vertical are 50 μm × 100 μm. In each row, two mirrored contacts with respect to the substrate axis are fabricated to double the number of devices per batch, while maintaining the isolation between adjacent structures (Fig. 2a). A lateral mask made from Nitto tape is manually applied to define the active channel area and protect the silver pads from being coated with MoS_2_ during the exfoliation step (Fig. 2b and h). This masking ensures that the electrodes remain exposed for later contact. However, the thickness of the Nitto tape (∼80 μm) sets a limit on the minimum size of the exposed window. In practice, the uncovered area must be at least 2 mm × 2 mm to guarantee conformal contact between the MoS_2_-coated tape and the SiO_2_ substrate during the transfer process.

Two Nitto tape strips are mounted on the rollers in Fig. 2c. The MoS_2_ flakes are first exfoliated with Scotch-tape method and placed on one of the Nitto strips. The rollers are rotated using an electric screwdriver to thin down and evenly distribute the MoS_2_ flakes across the adhesive surface. This process continues until the tape is uniformly covered, avoiding the presence of bulk material (Fig. 2g). The exfoliated MoS_2_ is then transferred onto the substrate (Fig. 2d and h) by applying a gentle pressure with tweezers, followed by heating on a hotplate for 5 minutes at 110 °C leading to a thermal release of the exfoliated nanosheets onto the acceptor surface. This step is repeated multiple times to ensure complete coverage of the whole BCs, to avoid pinholes between the TC and the BC. The MoS_2_ active layer is formed by 20 sequential transfers, each contributing a dense network of nanosheets. From related cross-sectional SEM investigations of sequentially transferred MoS_2_ films (to be reported separately), we found that each transfer typically adds 40–50 nm of thickness. Based on this, we estimate that the present devices employ an active layer in the 800–1000 nm range, as confirmed by a profilometer scan of the device area reported in Fig. S1. While the precise thickness is not critical for filamentary resistive switching, the reproducibility of the *I*–*V* characteristics across a large batch of devices indicates that the sequential transfer process ensures a continuous coverage of the active area of the devices, enabling stable operation. The morphology of the exfoliated MoS_2_ nanosheets obtained by the roll-to-roll process has been characterized in detail by AFM in our previous work,^31^ where a statistical analysis of nearly 200 flakes revealed a mean thickness of 40 nm and lateral sizes of a few to tens of micrometers. These dimensions are consistent with the 40–50 nm/transfer thickness inferred from cross-sectional SEM and confirm the suitability of the exfoliated flakes as building blocks for continuous active layers in memristive devices.

Once the middle area of the BCs is completely covered by the SL, the side mask is removed and the batch is cleaned in hot ACE (15 min at 40 °C), then rinsed in IPA and annealed on a hot plate at 110 °C for 20 minutes to remove any Nitto tape residues. Finally, the gold TC are printed on top of the stack (Fig. 2f and i), by heating the printer platen at 55 °C to enhance the wettability of the non-planar SL. Wider pads for the TC are printed outside the rough region, directly on SiO_2_ with silver ink, to improve the mechanical contact with the probe tip. At an early stage of this work, both contacts were printed using silver ink. The devices were observed to switch correctly into the LRS once the applied voltage exceeded *V*_SET_; however, they did not revert to the HRS even under large negative bias. This irreversible behavior is consistent with a switching mechanism based on the formation and break of metallic dendrites originating from the Ag electrode. In a symmetric structure, such directional filament dynamics are suppressed, preventing reliable RESET operations.

## 3 Results and discussion

### 3.1 Electrical characterization

**Fig. 3:**
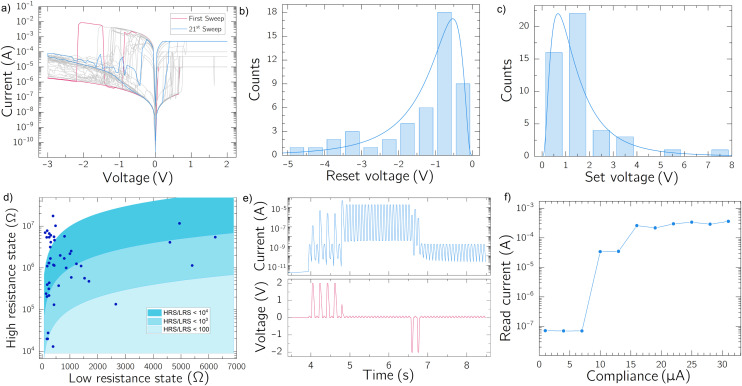
(a) Multiple Current–Voltage sweeps applied to a single devices, in red the first one and in blue the last. Different instrument current compliances (CCs) have been tested (b) RESET voltage measured for different devices, the statistical distribution was best fitted with a lognormal function with an average value (*μ*) of −0.025 and a standard deviation (*σ*) of 0.810 (c) SET voltages measured for different devices, the lognormal distribution in this case has *μ*: 0.202 and *σ*: 0.776 (d) 2D plot showing the resistance values for the two states for different devices. Three areas are highlighted depending on the resistance ratio between the two states, the majority of the devices has its states separated by at least 10^3^. (e) A read/write voltage waveform is applied to the device. 2 V/−2 V × 20 ms voltage pulses are the Write (SET/RESET) pulses and 100 mV × 20 ms are the Read pulses. (f) Read current after a SET voltage sweep. Consecutive sweeps have been applied at increasing instrument CC.

In Fig. 3a, we show the *I*–*V* characteristics of the two-terminal device, taken with a four step segment sweep: starting from zero voltage and increasing to the maximum set voltage, then decreasing back to zero, followed by a sweep to the minimum reset voltage, and finally returning to zero. During the first two segments, a fixed CC was applied to limit the growth of the CF and extend the device lifespan;^33^ several CCs have been tested, from 10 μA to 1 mA. In the third segment, the CC was removed, allowing the applied voltage to break the CF, thus, switching the device from the LRS to the HRS. Analysis of the voltage–current characteristics in linear scale provides insight into the switching mechanism (SI, Fig. S2). In the LRS, the current increases linearly with voltage, consistent with Ohm's law and indicative of metallic conduction through a conductive silver filament. By contrast, fitting the HRS curve yields a quadratic dependence (*I* ∝ *V*^2^), characteristic of Space Charge Limited Conduction (SCLC),^34^ in agreement with numerous reports in the literature.^26,32,35–39^

Four key device parameters were extracted from measurements: set voltage, reset voltage, resistance in the LRS and resistance in the HRS. The set and reset voltages (*V*_SET_ and *V*_RST_, respectively) correspond to the voltages at which the current changes abruptly, during the forwards and backwards sweeps, respectively. These values were obtained by calculating the derivative 
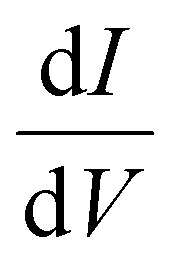
 and reading the voltages at which the minimum and maximum occurs (Fig. S3). The evolution of these parameters after consecutive sweeps is unpredictable as it can be seen in Fig. S4. However, since the digital logic only requires a clear distinction between the high- and low-resistive states, the precise control of the switching voltage and conductance is not needed and applying ±5 V would results in the correct SET/RESET of the device. Another important parameter is the endurance, that represent the maximum number of programming cycles before the failure of the device. The performed measurements reports that the maximum endurance for our devices is 20, after that the memristor switches permanently to the LRS (Fig. S5).

The histogram in Fig. 3b and c shows the distribution of *V*_SET_ and *V*_RST_ for the 47 functioning devices (with an average yield of 40% per batch). These values are significantly influenced by the SL thickness and the pre-form sweep. Following the statistical analysis, a global SET voltage of 5 V and a reset voltage of −5 V have been chosen to ensure a reliable execution of the writing operation. The high- and low-resistance states were determined by dividing the voltage of the first sample in the third sweep segment by the corresponding current, producing the scatter plot in Fig. 3d. Three main regions are highlighted, based on the ratio between the *x*-axis (LRS values) and the *y*-axis (HRS values), with each data point representing the two resistance values for different devices. The majority of memristors have a current ratio larger that 10^3^, with some even reaching 10^4^. The HRS values show larger dispersion, ranging from 10 kΩ to 100 MΩ. These two resistance states are further confirmed in the pulsed voltage test (Fig. 3e), where consecutive voltage pulses were applied until the current measured during a read pulse exceeded a predefined threshold value. The same procedure applies to the reset pulses. The applied pulses were 20 ms long and had an amplitude of 2/−2 V for the set/reset operation and 100 mV during the read. From these measures the switching speed can also be evaluated and it is estimated to span from 100 ms to lesser than 20 ms, depending on the amplitude and the CC of the instrument (Fig. S6).

Additionally, a series of consecutive SET sweeps were performed with progressively increasing CC followed by a read pulse to measure the current state, as shown in Fig. 3f. For very low compliances (*i.e.* 1 μA to 10 μA) the memristor exhibits a volatile data retention that turns into non-volatile when the CC is increased. In the first case, during the set sweep, it could be seen that the current abruptly switch from the HRS to the LRS, but the consecutive reading results in a low current, implying the loss of the write operation. The CC value to achieve the non-volatile state has a wide dispersion and sometimes it is not uniquely defined (Fig. S7), but it has been seen that for CC larger than 100 μA, the non volatile state was always achieved with data retention time longer than 10^3^ seconds (Fig. S8). However, increasing CC to the milliampere range led to a reduction in device lifespan, likely due to excessive CF growth, which prevented the device from reverting to the HRS.

In Table S1 (SI), we present a comparison of performance parameters across different memristive devices reported in the literature. The key contribution of this work lies in demonstrating a novel and fast fabrication method to realize ReRAM devices with competitive characteristics.

### 3.2 ReRAM simulation

From the previous measurements, the statistical distribution of the four key memristor parameters was evaluated to simulate their application as a ReRAM for storing the weights and biases of a fully connected neural network. The network's objective was to classify 5 × 4 pixel images representing schematic versions of the digits 0 to 9.

These simulations has been performed with the Tensorflow python package, building a sequential model with increasing number of neurons and weights to minimize the required bit-width for parameter storage.^40^ Since this package performs the backpropagation algorithm only with float32 datatype and does not allow to work with custom integer sizes, the approach proposed in this article consisted in a standard network training performed by the instruction model.fit() followed by a datatype conversion. Once the accuracy of the network reaches a value near 100% and the total loss is small, each layer variables were normalized to assume values ranging from −1 to 1, then multiplied the maximum value of the binary word (in C2 representation).

**Fig. 4:**
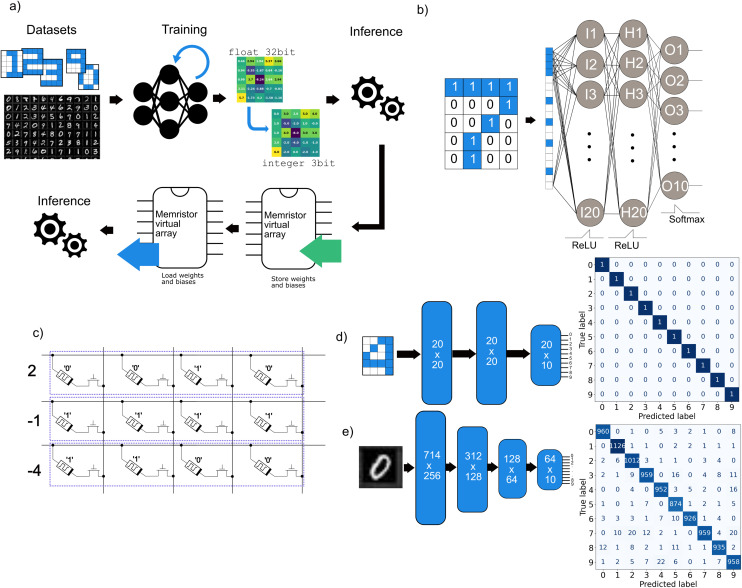
(a) Schematic of the performed simulation to train a neural network with post-train quantized weights and biases. (b) Schematic of the structure of the neural network implemented to recognize a simple dataset containing the digits from 0 to 9. (c) Memristors crossbar array, with an enable-transistor in series to access a single cell without sneak current paths. Using the crossbar array as a ReRAM, it is possible to store and load values for the parameters of the network in a two's complement. (d) Layout of the fully connected network used to recognize the simple dataset coded in 3 bits integers parameters with the corresponding confusion matrix obtained during inference. (e) Layout of the fully connected neural network used to recognize the MNIST dataset.

Simulation results have shown that a two-layer network with 10 neurons per layer could successfully classify all images in the dataset, even after weights and biases were quantized to integer formats ranging from 32-bit down to 8-bit. However, at 4-bit precision, accuracy degradation became evident, requiring the addition of new layers. A three-layer fully connected network (Fig. 4d) achieved 100% accuracy, with input and hidden layers consisting of 20 neurons each, and an output layer containing 10 neurons (Fig. 4b).

The trained parameters were then mapped onto a simulated memristor array, where each cell was implemented as a Python class containing four key parameters selected randomly from their measured distribution. Each virtual memristor had unique resistance states and set/reset voltages and represented a single-bit weight. As a result, the memristor array size was three times larger than the original neural network parameter array. Writing or resetting each cell was simulated by applying an appropriate voltage, chosen after the analysis of the evolution of *V*_SET_ and *V*_RST_ to its terminals. To evaluate robustness, noise was introduced during inference by flipping a random pixel in each digit of the dataset. Due to the small size of the dataset (10 images), the noise has reduced the classification accuracy (Fig. S9) but it heavily depends on the flipped pixel, resulting in 100% accurate prediction down to 70%. The impact strongly depended on which pixel was corrupted, as a single flip could effectively transform a digit into something visually close to another, justifying the resulting misclassifications.

Then, the network has been extended to classify the more complex MNIST dataset with the same approach illustrated previously (Fig. 4e). The network has been first trained with all parameters in the float32 format, and then the weights have been quantized into a representation with decreasing number of bits. It has been observed that a network with four fully connected layers, *i.e.*, an input, two hidden and an output layer with 10 neurons, provides an accuracy near 97% when its variables are converted from floating point to 4 bit signed integer. Increasing the number of neurons or layers does not enable the network to achieve similar classification accuracy with fewer bits per weight. Nonetheless, a simple memristor crossbar array provides a feasible means to store weights and biases.

## Conclusions

In this work, Ag/MoS_2_/Au memristors were fabricated using a high-throughput, low-cost approach, combining mechanical exfoliation and inkjet printing to define the switching layer (SL) and metal electrodes, respectively. Electrical characterization have shown abrupt resistance switching in the fabricated devices, likely governed by the formation and dissolution of silver conductive filaments across MoS_2_ layer. The devices exhibited a resistance ratio ranging from 10^2^ to 10^4^ and retention times exceeding 10^3^ seconds, confirming their suitability for non-volatile memory applications.

To explore their potential for neuromorphic computing, the fabricated devices were modeled in a ReRAM-based crossbar array to store neural network parameters. A simple digit recognition task was implemented, demonstrating that three-bit integer quantization of network weights and biases did not degrade accuracy. However, to achieve over 90% accuracy on a larger neural network for MNIST dataset classification, at least four-bit parameter quantization is required.

These findings suggest that 2D-material-based memristors are viable candidates for non-volatile memory and neuromorphic computing applications. Future work will focus on the physical integration of access transistors to enable large-scale crossbar arrays and the exploration of multi-level resistance states by tuning compliance currents during the SET process. If these memristors can reliably achieve multiple resistance levels, they could be leveraged to enable a direct in-memory implementation of vector-matrix multiplication, accelerating neuromorphic and AI-driven computations.

## Conflicts of interest

There are no conflicts to declare.

## Supplementary Material

NR-018-D5NR02690C-s001

## Data Availability

Data for this article, including all excel files used for statistics and characterization will be available at Zenodo [https://doi.org/10.5281/zenodo.17481788], the python code written to perform the simulation and the neural network is available on GitHub [https://github.com/Gmarraccini/ReRAM] and Google Colab for the 4 bit Network used for the classification of the MNIST dataset [https://colab.research.google.com/drive/18qYdLm4wybe9RAYBI324uCeWs2JhDxpQ?usp=sharing]. Supplementary information (SI) is available. See DOI: https://doi.org/10.1039/d5nr02690c.
