# Supplementary material for: Fast prototyping of memristors for ReRAMs and neuromorphic computing
Source: Nanoscale. 2025 Dec 10;18(2):835–42. doi: 10.1039/d5nr02690c (PMC12690548; doi:10.1039/d5nr02690c)
Supplement: NR-018-D5NR02690C-s001 [file NR-018-D5NR02690C-s001.pdf]

Supplementary Information (SI) for Nanoscale.
This journal is © The Royal Society of Chemistry 2025

REPORT NUMBER

High-throughput fabrication of 2D MoS_2-based memristors for Neuromorphic computing
Supporting Information

Gianluca Marraccini,[1,2] Sebastiano Strangio,[1] Elisabetta Dimaggio,[1] Riccardo Sargeni,[1,3] Francesco Pieri,[1] Yigit Sozen,[4] Andres Castellanos Gomez,[4] and Gianluca Fiori[1]

[1] *Dipartimento di Ingegneria dell'Informazione, Università di Pisa, Pisa, Italy*

[2] *Department of Electrical, Computer and Biomedical Engineering, Università di Pavia, Pavia, Italy*

[3] *Quantavis s.r.l., Largo Padre Renzo Spadoni, Pisa, Italy*

[4] *2D Foundry research group. Instituto de Ciencia de Materiales de Madrid (ICMM-CSIC), Madrid, E-28049, Spain.*

(Dated: October 6, 2025)

SWITCHING LAYER

Following the fabrication procedure described in Section 2, the Switching Layer (SL) thickness was measured. The samples were produced with an increasing number of transfer steps. Since most devices in the batch continued to exhibit pre-short-circuit behavior, the number of layers was set to 20 in order to mitigate this issue. Increasing the number of transfers is expected to reduce the presence of pinholes, thereby lowering the probability of device failure. Although up to 25 consecutive transfers were tested, no further improvement in device yield was observed beyond 20 layers.

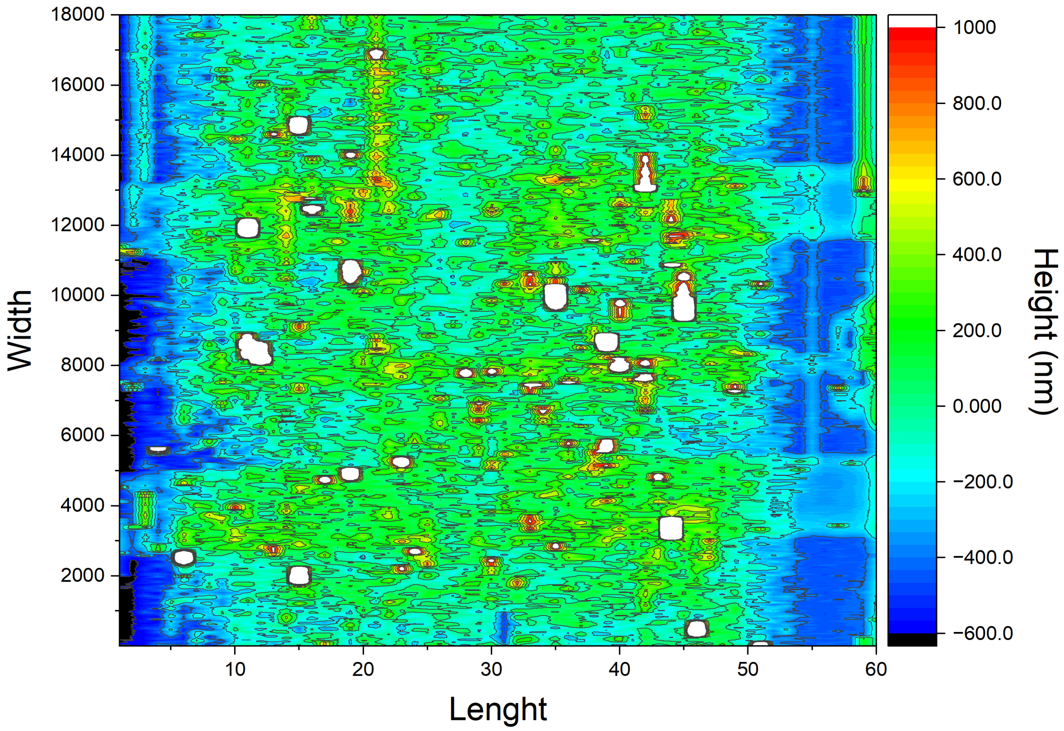

Figure 1. Thickness profile taken with a Bruker profilometer. On the x-axis there is the horizontal dimension and on the y-axis the respective height.

CONTACT RESISTANCE

To gain insight into the switching mechanism of the proposed devices, we analyzed the electrical measurements by fitting the sperimental data with a linear curve ($I = V \cdot G$ for the low resistive state (LRS)) and with a power function of the voltage ($I = a \cdot V^b$ for the high resistive state (HRS)). This can be interpreted as a metallic behaviour in the former and a bulk-limited conduction in the latter voltage regions.

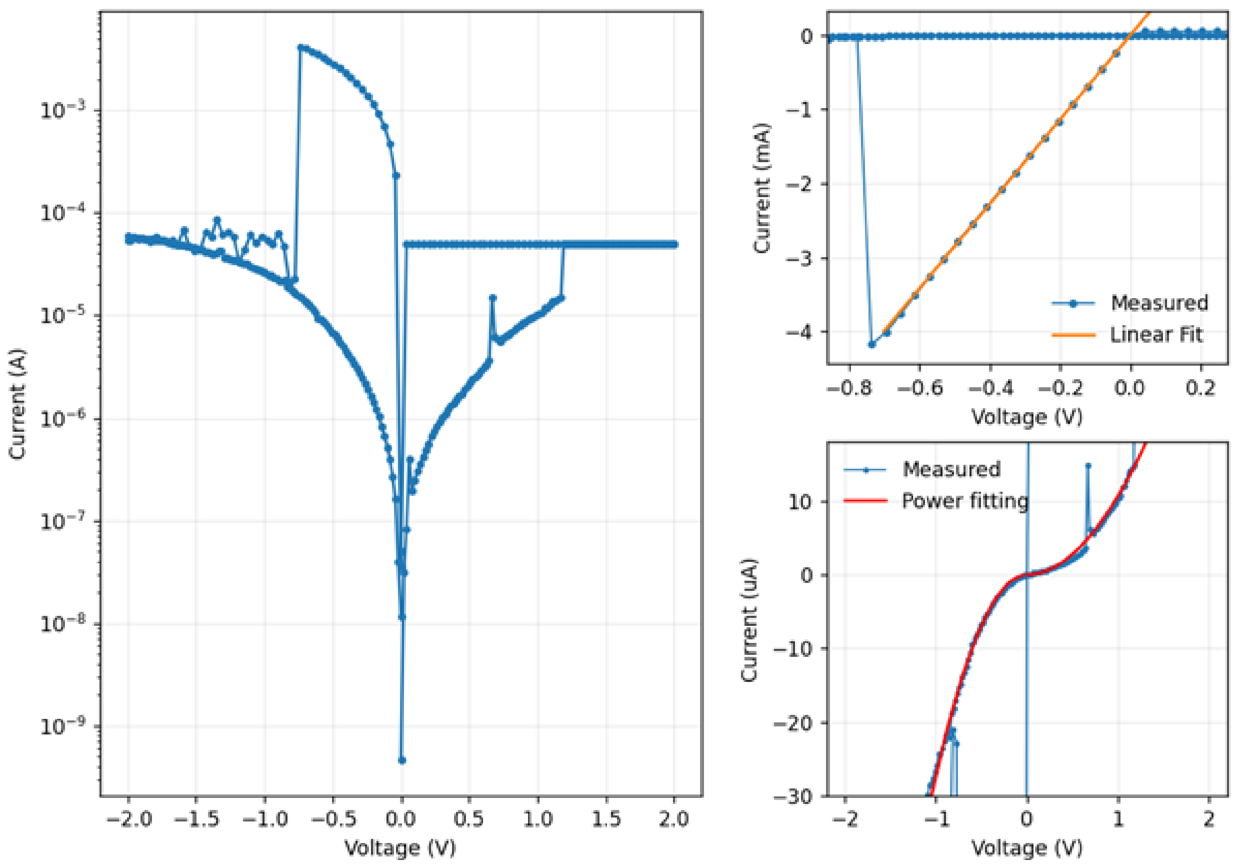

Figure 2. Current-Voltage relationship analysis a)A semi-logarithimic plot of the absilute value of the current verus voltage b) Linear scale graph picturing a linear relationship between the current and voltage, confirmed by the linear fit. c) Linear graph zoomed in the High Resistance State. As it can be seen by the red curve, the fitted curve match the sperimental data when the power function $I = A \cdot V^b$ is used. In particular, the parameters that fitted the plotted curve are $I = 1.069 \cdot V^{1.939}$ for the positive voltage and $I = -2.735 \cdot V^{1.982}$ for the negative voltages, coherently with the Space Charge Limited Current (SCLC)

PARAMETERS EXTRACTION

By postprocessing the I-V characteristics of the devices, as the one shown on the left of Figure 3, four characteristic parameters were calculated. The voltage at which the current abruptly rises is the set voltage (V_{SET}), the voltage at which it falls is the reset voltage (V_{RST}). Both parameters are extracted from the derivative $\frac{\partial I}{\partial V}$ of the I-V characteristic (shown on the right of Figure 3) and are the voltages at which the positive and negative conductance spikes occur, respectively.

The resistance values of the high and low states are calculated as the $\frac{V}{I}$ ratio at:

- V= 50 mV and the corresponding current value, for the High Resistance State;
- V= -50 mV and the corresponding current value, for the the Low Resistance State.

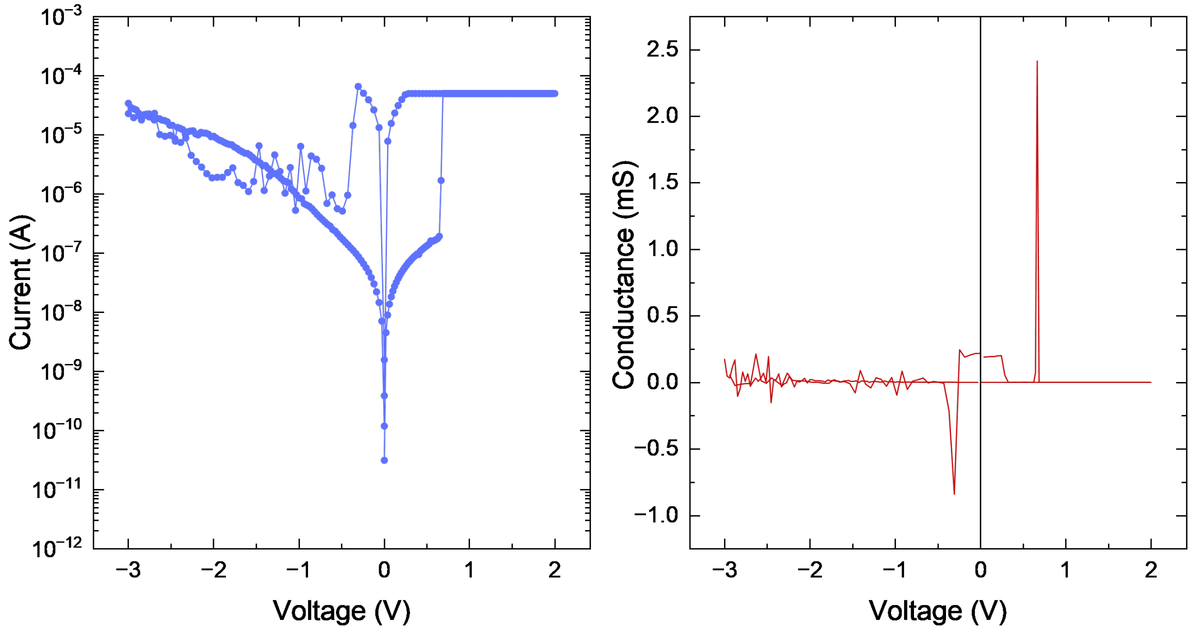

Figure 3. On the left: I-V characteristic of a measured memristor. On the right: Extracted conductance values from the former graph. The discrete derivative of I over V has been applied to the data. The SET and RESET peaks can be seen, while the conductance value is the metal contact one near the 0V and is 0 when the instrument CC is active.

SET/RESET VOLTAGES

To determine a sufficient voltage capable of setting every device in the virtual crossbar array, the evolution of the Set and Reset voltages was analyzed over consecutive sweeps. There is high variability among devices and measurements, nonetheless, the maximum and minimum V_{SET} and V_{RST} remain in a stable range of voltages. For the statistical analysis and the virtual simulation of the crossbar array, the maximum value of V_{SET} and the minimum value of V_{RESET} were selected among the set and reset sweeps, respectively, to ensure reliable execution of the writing operation. A global SET voltage of 5 V and a reset voltage of -5 V have been chosen.

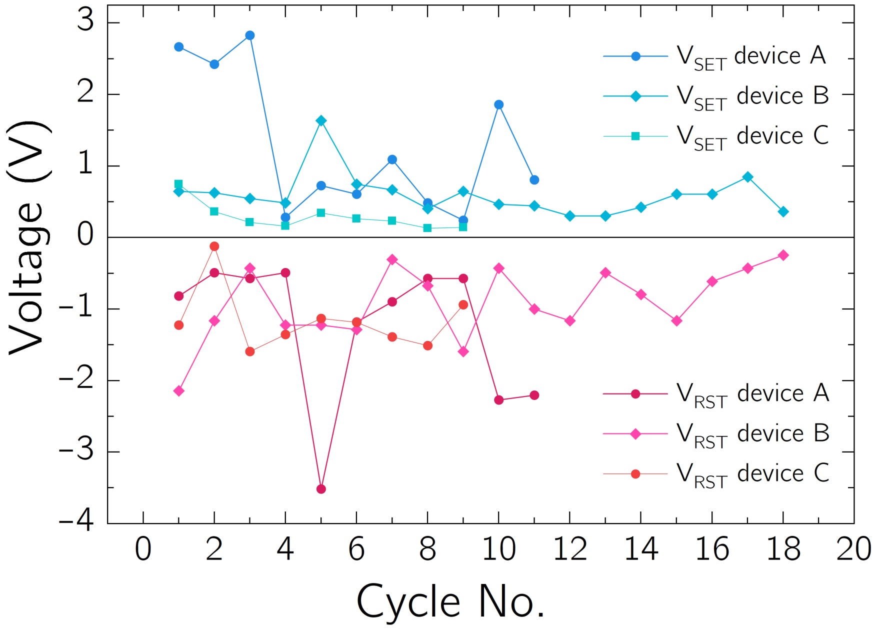

Figure 4. Plot of the extracted Set and Reset voltages from consecutive voltage sweep in a single device, obtained with the previously described method.

ENDURANCE

An important parameter for evaluating device robustness is endurance, defined as the maximum num-ber of SET/RESET cycles that the memristor can reliably undergo before one of the two operations fails This metric represents the number of times the memory can be reprogrammed while still ensuring cor-rect data storage. In the present devices, the average endurance is approximately 20 cycles before the memristor becomes permanently stuck in the low-resistive state (LRS). Although this value is lower than those reported in other works, the envisioned application—storage of neural network weights—would require only a single programming cycle, since training and quantization are carried out ex situ.

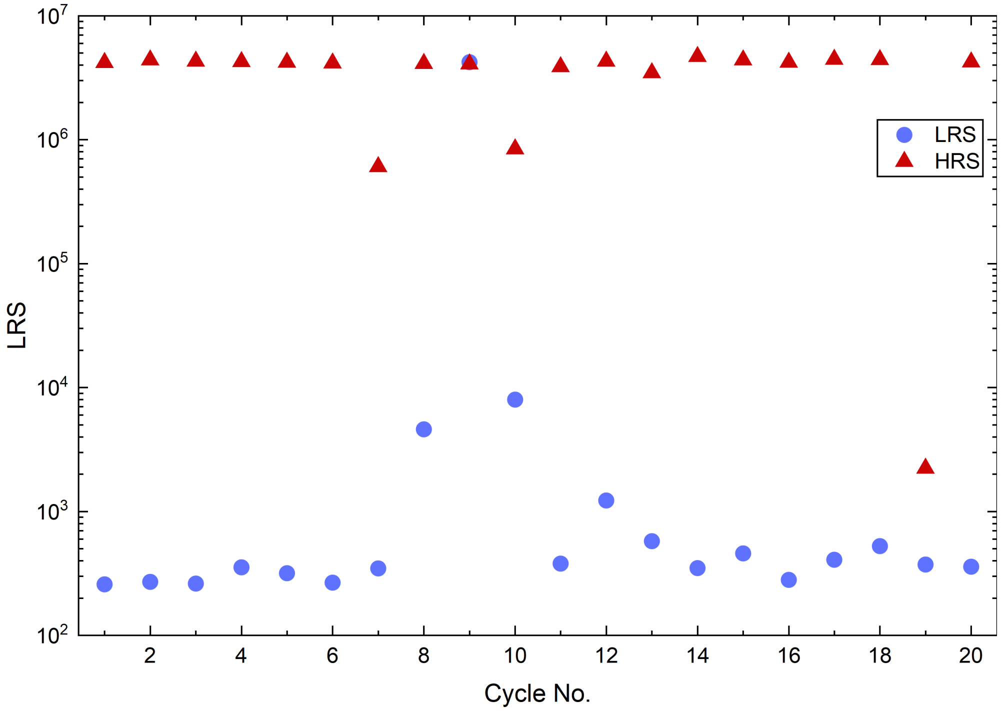

Figure 5. Consecutive V-I sweeps performed at fixed compliances ($200\mu A$) in order to evaluate the number of cycles of a device before switching irreversibly to the LRS.

SWITCHING SPEED

In this section, we present transient measurements of the SET and RESET operations. A sequence of voltage pulses was applied to the device terminals to consecutively switch between the two states and measure the corresponding switching times. Figure 6-top shows two complete SET/RESET cycles with intermediate read operations. To ensure reliable switching, the SET pulse was 500 ms long and 3 V high, limited by an instrument compliance of 200 μA. The RESET pulse was 800 ms long at -5 V and uncapped. The read voltage was set to 50 mV for 20 ms.

Figure 6-bottom shows a faster pulse train for SET/RESET and read operations. In this case, since the current compliance was fixed at the empirical limit of 100 μA for non-volatile switching, the SET operation was not immediate but instead required multiple pulses to complete. The switching time is strongly dependent on both the current compliance and the pulse amplitude.

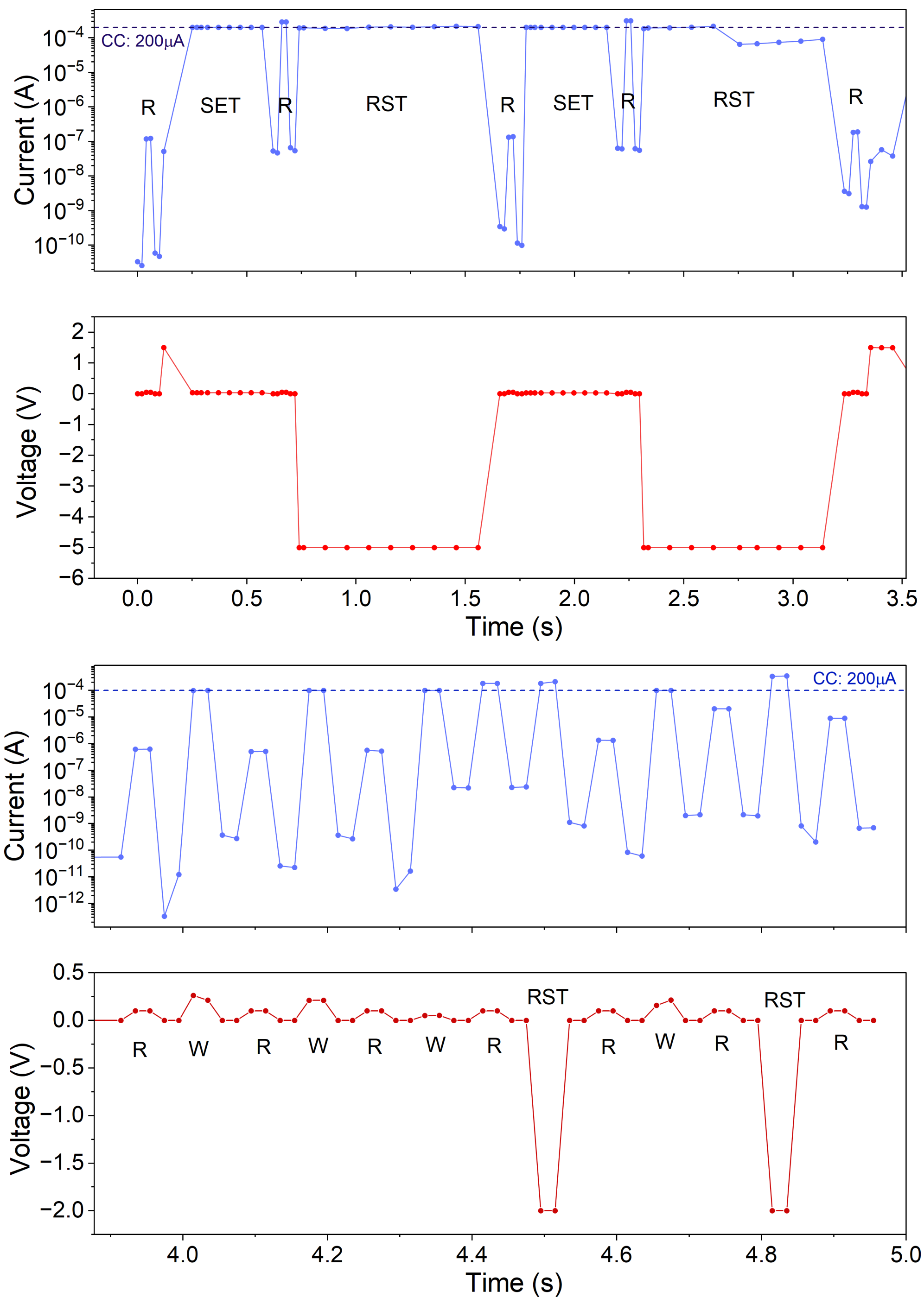

Figure 6. Two transient measures applying different voltage pulses to the devices Top) 2V x 0.5s SET pulse and -5V x 1s RESET pulse, between them there is a read operation at 50mV for 20ms. The set operation occurs in the first 100ms from the start of the pulse while the reset is faster due to the high voltage applied. Bottom) A faster pulse train with consecutive write pulses, but with a fixed instrument current compliance. It can be seen that the SET operation is achieved after four pulses due to the fast speed of the pulses and the current low current compliance.

CURRENT COMPLIANCE ANALYSIS

The effect of the applied instrument current compliance on the resistance state was studied, by applying different positive voltage sweeps to the device, and varying the current compliance parameter from 10 to 100 μA. The parametric voltage sweeps are shown in Figure 7a. In order to measure the correct SET operation, i.e. the transition from the high resistance state to the low resistance state, the current was red after each sweep with a reading voltage of 100 mV applied to the device. When the current compliance was above 70-80 μA, the SET operation was always successful (as shown in Figure 7b), with a correct transition to the low resistance state and no conductance modulation observed. For lower compliancies, instead, the operation gave a volatile data retention, as confirmed by the results on three different devices reported in Figure 7c, where the presence of two resistance states is confirmed, but with an unstable switch between them.

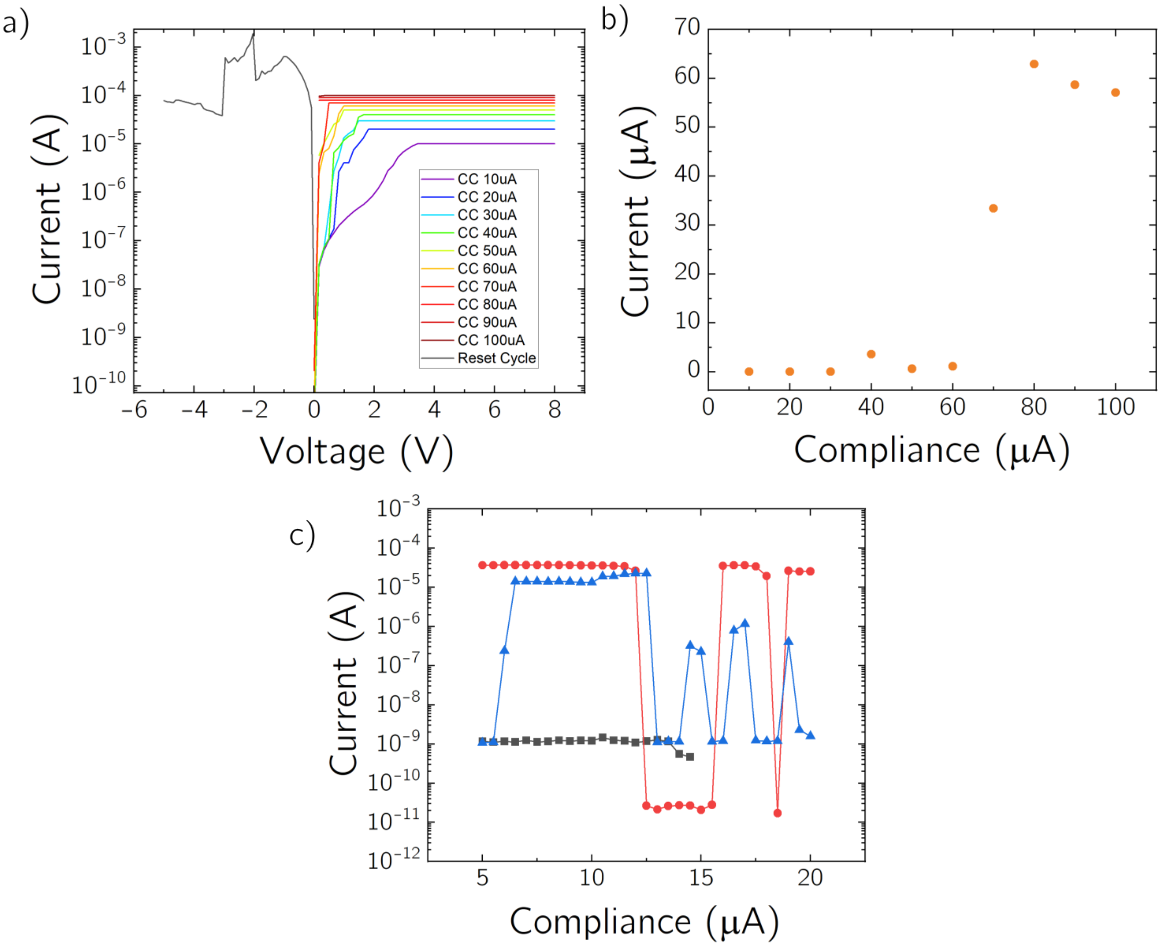

Figure 7. a) Consecutive positive voltage sweeps from 0 to 8V applied to the same device, increasing the instrument current compliance from 10 to 100 μA. After each sweep, a current is measured at the reading voltage of 100 mV. Then a negative sweep is applied to reset the device in the High Resistive State. b) Measured current after each sweep versus the instrument current compliance. It can be seen that only two states are achieved, and, at compliancies lower than 80 μA, the device shows a volatile data retention. c) Focus on the low compliance region(5-20 muA): the SET sweep does not always change the device resistance state.

RETENTION TIME

In order to measure how long the device would retain the Low Resistance State, transient measurements have been performed at the reading voltage of -50 mV, until the devices reverted back to the High Resistance State, see Figure 8. All the devices showed stable current values for 1200 seconds, then, the majority of devices became unstable or switched the resistance state.

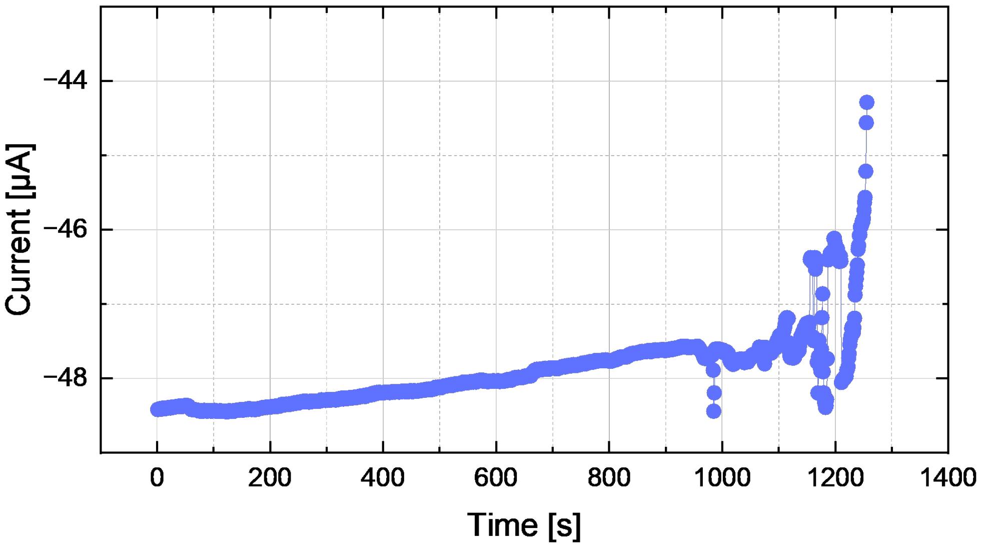

Figure 8. The sweep cycle set with a current compliance of 1 mA, the read voltage is -50 mA. The data is retained for 1200 seconds. Then, the state of the memristor switched back to the high resistance state (HRS).

MEMRISTOR BENCHMARK IN LITERATURE

To summarize the results obtained in this work, the key performance parameters are benchmarked against those of memristors reported in the literature. The comparative table below highlights similarities and differences in terms of device structure, fabrication approach, and switching characteristics, thereby placing the present results within the broader context of resistive memory research.

**Article**	**Structure**	**Fabrication**	**Active Area (μm^2)**	**Endurance (No. Cycles)**	$V_{SET}(V)$	$V_{RST}(V)$	$\frac{I_{ON}}{I_{OFF}}$	**Retention Time (s)**	**Switch Time**	**Conductance states**
This work	Ag/MoS_2/Au	ME/IJP	50×50	20	3	-2	10^3	1.2×10^3	20ms	2
[1]	Ag/MoS_2/ MoO_x/Ag	LPE/IJP	130×170	10^4	0.2	-0.1	10^6	10^4	100 ms	Long/short Time E/D
[2]	Ag/hBN/Pt	LPE	NR	6×10^5	0.4 to 16	-	10 to10^5	$> 10^3$	1 ms	-
[3]	Pt/MoS_2/Ti	LPE/Spin coating	NR	10^7	0.65	-0.9	10^5	10^5	40 ns	10
[4]	Cu/MoS_2 /Au	Lithography	2×2	NR	0.25	-0.15	10^3	2×10^4	NR	Analog E/D
[5]	Ag/WSe_2/Ag	AJP	70×70	90	0.5	0.3	10^3	10^4	700ns	2
[6]	Ag/hBN/Cu	CVD/ Lithography	NR	550	0.72	-0.37	100	3×10^3	NR	2
[7]	Ag/MoS_2/Ag	AJP	100×100	NR	0.18	-0.1 to -0.8	10^5	4×10^4	70 ns	
[8]	Au/Ti/ hBN/Cu	CVD/ Lithography	5×5 to 150×150	500	2	-2		3.3×10^3	10 ns	Short Time Depression
[9]	Ag/HKUST -1/ITO	IJP/ Crystallization	< 2500	NR	1.45	-0.8	10^4	600	NR	2
[10]	Ag/SOG/ PEDOT:PSS	IJP/spin coating	100×100	1100	0.6	<0.1	10^4	1.7×10^4	NR	2
[11]	Ag/MoS_2/Au	ME/ Lithography	5×5	>100	0.01	-0.04	5	10^4	NR	10
[12]	Au/MoS_2/Au	ME/ Lithography	2×5	>20	4	-	10^3	3×10^3	-	Light controlled STE/LTP
[13]	Gr/MoS_2/ Gr	Litho- graphy/ME	NR	2×10^7	3.5	-4.8	100	10^5	100ns	2

Table I. Comparative table reporting the main parameter for different memristor works found in literature, form simple memories to synapses. ”NR”: Not Reported, ”-”: Not applicable

CORRUPTED DATASET WITH THE 3-BIT INTEGER NETWORK

In order to test the strength of the 3 layer - 3 bit integer neural network, after measuring the accuracy by performing the inference with the same simple dataset used in the training, a corrupted version of the same dataset, shown on the left of Figure 9, was used, where one random pixel per digit has been flipped. The matrix on the right of Figure 9 shows that the accuracy heavily depends on the position of the introduced noise, since it can distort a digit into another due to the low resolution of the images.

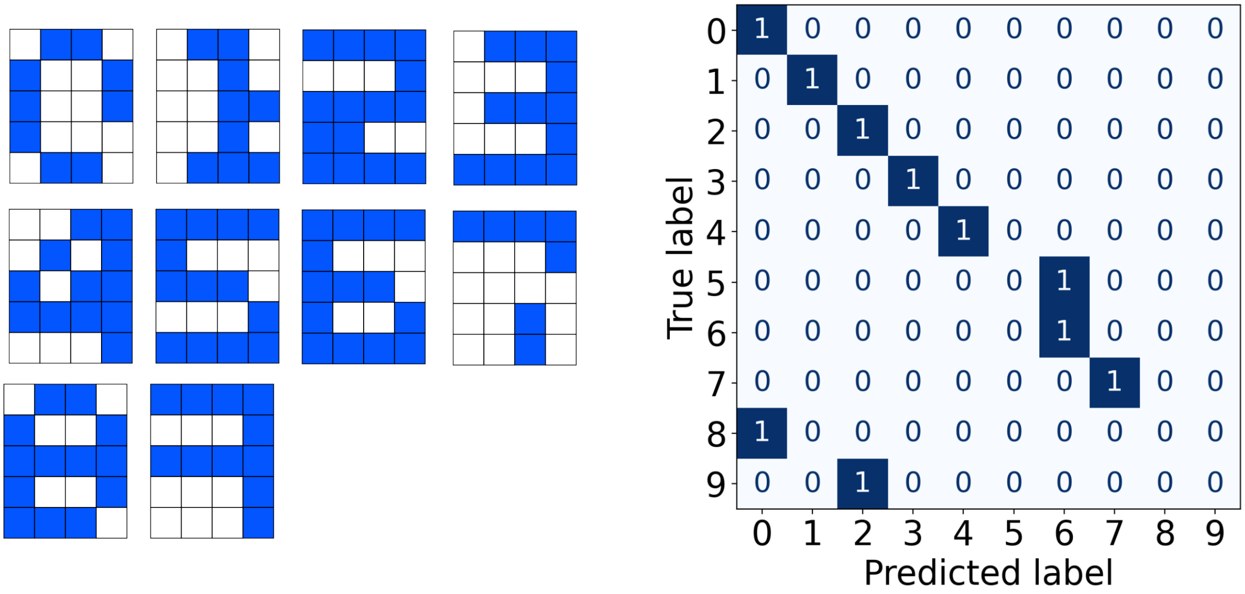

Figure 9. The corrupted toy-dataset (on the left) has been obtained with a simple script that invert a pixel of the 5 × 4 randomly. As it can be seen from the figure on the right, the accuracy decreases significantly due to the little dataset during the training. The noise effect on the images can be mitigated choosing digits images more different from each other.

REFERENCES

[1] Bessonov, A., Kirikova, M., Petukhov, D. et al. Layered memristive and memcapacitive switches for printable electronics. Nature Mater 14, 199–204 (2015). https://doi.org/10.1038/nmat4135

[2] Kaichen Zhu et al. "Inkjet-printed h-BN memristors for hardware security" Nanoscale, 2023,15, 9985-9992. https://doi.org/10.1039/d3nr00030c

[3] Tang, B., Veluri, H., Li, Y. et al. Wafer-scale solution-processed 2D material analog resistive memory array for memory-based computing. Nat Commun 13, 3037 (2022). https://doi.org/10.1038/s41467-022-30519-w

[4] Vertical MoS2 Double-Layer Memristor with Electrochemical Metallization as an Atomic-Scale Synapse with Switching Thresholds Approaching 100 mV. Renjing Xu, Houk Jang, Min-Hyun Lee, Dovran Amanov, Yeonchoo Cho, Haeryong Kim, Seongjun Park, Hyeon-jin Shin, and Donhee Ham, Nano Letters 2019 19 (4), 2411-2417, DOI: 10.1021/acs.nanolett.8b05140

[5] Sivan, M., Li, Y., Veluri, H. et al. All WSe2 1T1R resistive RAM cell for future monolithic 3D embedded memory integration. Nat Commun 10, 5201 (2019). https://doi.org/10.1038/s41467-019-13176-4

[6] Qian, K., Tay, R.Y., Nguyen, V.C., Wang, J., Cai, G., Chen, T., Teo, E.H.T. and Lee, P.S. (2016), Hexagonal Boron Nitride Thin Film for Flexible Resistive Memory Applications. Adv. Funct. Mater., 26: 2176-2184. https://doi.org/10.1002/adfm.201504771

[7] X. Feng, Y. Li, L. Wang, S. Chen, Z. G. Yu, W. C. Tan, N. Macadam, G. Hu, L. Huang, L. Chen, X. Gong, D. Chi, T. Hasan, A. V.-Y. Thean, Y.-W. Zhang, K.-W. Ang, A Fully Printed Flexible MoS2 Memristive Artificial Synapse with Femtojoule Switching Energy. Adv. Electron. Mater. 2019, 5, 1900740. https://doi.org/10.1002/aelm.201900740

[8] Shi, Y., Liang, X., Yuan, B. et al. Electronic synapses made of layered two-dimensional materials. Nat Electron 1, 458–465 (2018). https://doi.org/10.1038/s41928-018-0118-9

[9] Y. Liu, F. Fischer, H. Hu, H. Gliemann, C. Natzeck, M. Schwotzer, C. Rainer, U. Lemmer, C. Wöll, B. Breitung, J. Aghassi-Hagmann, Inkjet Printed Metal–Organic Frameworks for Non-Volatile Memory Devices Suitable for Printed RRAM. Adv. Funct. Mater. 2025, 35, 2412372. https://doi.org/10.1002/adfm.202412372

[10] B. Huber, P. B. Popp, M. Kaiser, A. Ruediger, C. Schindler; Fully inkjet printed flexible resistive memory. Appl. Phys. Lett. 3 April 2017; 110 (14): 143503. https://doi.org/10.1063/1.4978664

[11] Zhang, Ziyi & Zhu, Xiaojian & Wang, Lixun & Ye, Xiaoyu & Gao, Runsheng & Zhang, Yuejun & Li, Run-Wei. (2025). Quantized conductance in MoS2 memristors for high-accuracy neuromorphic computing. Journal of Physics D: Applied Physics. 58. 10.1088/1361-6463/adcfae.

[12] Free-Standing Multilayer Molybdenum Disulfide Memristor for Brain-Inspired Neuromorphic Applications. Amin Abnavi, Ribwar Ahmadi, Amirhossein Hasani, Mirette Fawzy, Mohammad Reza Mohammadzadeh, Thushani De Silva, Niannian Yu, and Michael M. Adachi ACS Applied Materials & Interfaces 2021 13 (38), 45843-45853 DOI: 10.1021/acsami.1c11359

[13] Wang, Miao, et al. "Robust memristors based on layered two-dimensional materials." Nature Electronics 1.2 (2018): 130-136.
